# Biomimetic Tendon-Based Mechanism for Finger Flexion and Extension in a Soft Hand Exoskeleton: Design and Experimental Assessment

**DOI:** 10.3390/s23042272

**Published:** 2023-02-17

**Authors:** Mohamed H. Abdelhafiz, Lotte N. S. Andreasen Struijk, Strahinja Dosen, Erika G. Spaich

**Affiliations:** 1 Neurorehabilitation Systems Group, Department of Health Science and Technology, Aalborg University, 9260 Gistrup, Denmark; 2 Neurorehabilitation Robotics and Engineering Group, Center for Rehabilitation Robotics, Department of Health Science and Technology, Aalborg University, 9260 Gistrup, Denmark

**Keywords:** hand exoskeleton, assistive devices, rehabilitation devices, tendon-based systems

## Abstract

This study proposes a bioinspired exotendon routing configuration for a tendon-based mechanism to provide finger flexion and extension that utilizes a single motor to reduce the complexity of the system. The configuration was primarily inspired by the extrinsic muscle–tendon units of the human musculoskeletal system. The function of the intrinsic muscle–tendon units was partially compensated by adding a minor modification to the configuration of the extrinsic units. The finger kinematics produced by this solution during flexion and extension were experimentally evaluated on an artificial finger and compared to that obtained using the traditional mechanism, where one exotendon was inserted at the distal phalanx. The experiments were conducted on nine healthy subjects who wore a soft exoskeleton glove equipped with the novel tendon mechanism. Contrary to the traditional approach, the proposed mechanism successfully prevented the hyperextension of the distal interphalangeal (DIP) and the metacarpophalangeal (MCP) joints. During flexion, the DIP joint angles produced by the novel mechanism were smaller than the angles generated by the traditional approach for the same proximal interphalangeal (PIP) joint angles. This provided a flexion trajectory closer to the voluntary flexion motion and avoided straining the interphalangeal coupling between the DIP and PIP joints. Finally, the proposed solution generated similar trajectories when applied to a stiff artificial finger (simulating spasticity). The results, therefore, demonstrate that the proposed approach is indeed an effective solution for the envisioned soft hand exoskeleton system.

## 1. Introduction

The hand is essential for humans to interact with the environment, as it provides the ability to grasp, sense, and manipulate objects of different shapes and sizes. However, millions of people around the world suffer from hand motor impairments due to several neuromuscular diseases and injuries [1]. For example, up to 80% of stroke survivors experience neurological deficits, leading to upper limb impairment [2,3]. Weakness, spasticity, and abnormal muscle synergies are typical symptoms resulting in a significant reduction in the number of activities performed daily [4], which consequently decreases the quality of life [5]. Therefore, rehabilitating the upper limb, including the hand, is considered one of the top three priorities by stroke survivors and their caregivers [6]. Hand rehabilitation devices like Gloreha [7] and MIT-MANUS [8] have proven their efficiency in reducing hand impairment for acute stroke patients, however, their use is confined to clinics. Therefore, to further improve the outcome of hand rehabilitation, therapy should not be limited to training sessions in a hospital. This has motivated technology developers to design wearable active hand orthoses that can facilitate unsupervised training outside the clinics. Increasing the wearability and portability of these active hand orthoses has broadened the scope of their purposes to include assisting patients to accomplish activities of daily living. The purpose of the use of the orthoses has a significant impact on the design and functionality of the device, specifically on the power transmission mechanism, actuator selection, and human–machine interaction [9]. Developers of hand orthoses for assistive purposes usually focus on optimizing the size and the weight of the actuator and the transmission method of the device to produce an overall compact and light apparatus. For developers of orthoses for rehabilitation purposes only, the size and weight features of the device are instead usually not considered a priority compared to the ability to provide precise finger control. In the literature, several efforts to develop dual-purpose orthoses for both hand assistance and rehabilitation were reported [10].

To reduce the size of the hand exoskeleton and increase its wearability and portability, soft exoskeletons based on artificial tendons have been proposed [11]. These systems use a soft glove with exotendons inserted at the fingers to transmit the power from an actuator, which can be located away from the hand. Carbonhand^®^ [12] and the glove proposed by Liarokapis et al. [13] are exotendon-based gloves with a finger flexion mechanism to provide a strong grasp. The flexion mechanism in these gloves depends on one exotendon for each finger, which passes through sheaths across the finger phalanges to be inserted on the distal phalanx. The literature showed that this traditional exotendon path for finger flexion, where the exotendon is inserted at the distal phalanx, may provide an abnormal finger motion since it produces an incorrect bending sequence for the finger joints, where the distal joints of the finger have a higher priority in the moving sequence than the proximal joints [14]. The ASR-Glove [15] and FLEXotendon glove [16] present a two DoF flexion system actuated via tendons for each finger. In these gloves, one end of the tendon was fastened to the proximal phalanx to flex it individually, while the other end was inserted into the distal phalanx similarly to the traditional configuration mentioned earlier.

The Exo-Glove [17], Exo-Glove Poly II [18], HERO glove [19], and the glove proposed by Xiloyannis et al. [20] support finger extension, in addition to flexion. In these gloves, the extension mechanism consisted of one exotendon for each finger running along the phalanges to be inserted at the distal phalanx. This method for extending the finger might result in users experiencing finger joint hyperextension [19,21]. Therefore, the SPAR glove [22] used a hybrid design that incorporates rigid and soft elements to prevent the hyperextension of the finger joints. Dong Hyun Kim et al. [23] proposed an extension mechanism, fully based on exotendons, inspired by the extrinsic and intrinsic muscle–tendon units (i.e., extensor digitorum communis (EDC) and lumbrical muscles, respectively) to provide a natural finger motion and balance between the proximal interphalangeal (PIP) and metacarpophalangeal (MCP) joints during extension. The BiomHED glove [24] presented a bioinspired closing and opening mechanism based on replicating the extrinsic EDC and flexor digitorum profundus (FDP) and intrinsic tendons (radial and ulnar interosseous). Each of these tendons requires an exotendon and an actuator to replicate its function.

In general, tendon-based gloves increased the wearability and portability of hand exoskeletons, however, they could affect the ability to perform normal finger movements as they usually employ under-actuated mechanisms [14]. Bioinspired approaches, where the function of the extrinsic and intrinsic tendons was mimicked, improved this ability at the expense of increasing the number of tendons required to control the movement of the finger [24], which consequently might increase the complexity of the overall system. This study presents a bioinspired exotendon routing configuration for soft hand exoskeletons. The proposed configuration is designed to be simple, with only one actuator required to activate the finger in both the flexion and extension directions. To achieve this simple design, only the extrinsic tendons were mimicked while the function of the intrinsic tendon was partially compensated by adding minor modifications to the configuration of the extrinsic tendons. The proposed design is expected to provide close to normal finger flexion, where the phalanges flex simultaneously and not in sequence, and to prevent joint hyper-extension. The proposed exotendon routing configuration was assessed experimentally on one finger to evaluate its ability to support natural finger flexion and extension. This design has the potential to be implemented for multiple fingers on soft robotics orthoses for hand rehabilitation and assistance.

## 2. Design Concept and Prototype

**Figure 1:**
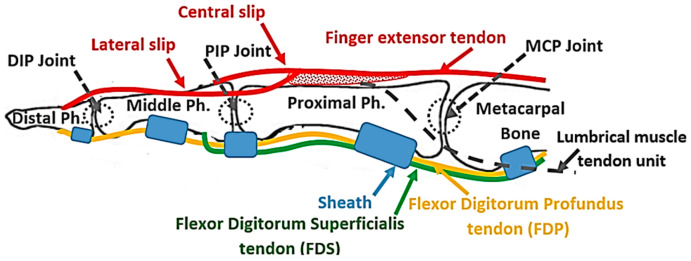
Anatomic structure of the finger with the flexor digitorum profundus (FDP) tendon and flexor digitorum superficialis (FDS) tendon (yellow and green lines, respectively), and extensor tendon (red line). Tendon sheets are presented in blue.

In the human finger, the distal interphalangeal (DIP) and the PIP joints, as well as the MCP joint, are extended using the EDC extrinsic muscle–tendon unit. The EDC muscle–tendon unit is attached proximally to the forearm and the tendon runs along the dorsal side of the hand and the finger to be separated at the proximal phalanx into three slips, namely, a central slip and two lateral slips (Figure 1). The central slip is inserted at the proximal side of the middle phalanx, while the lateral slips run along the ulnar and radial sides of the PIP joint and reunite on the dorsal side of the middle phalanx, to be inserted on the distal phalanx (Figure 1). When the PIP joint extends, the lateral slip of EDC tightens and pulls the distal phalanx, extending the DIP joint [25]. This structure kinematically couples the interphalangeal joints, making the extension of the DIP joint too dependent on the extension of the PIP joint, as demonstrated through mathematical modeling by Spoor et al. [25]. This coupling is named PIP–DIP interdependence [26]. The MCP joint extends under the effect of the force transmitted by the EDC tendon that is attached to the distal and proximal phalanges [27], as described earlier. The torque, generated by the EDC tendon force at the distal and proximal phalanges, is transmitted from one segment to another through the kinetic chain until it reaches the MCP joint.

Finger flexion is achieved by the action of the flexor digitorum superficialis (FDS) and the FDP extrinsic muscles. The FDS tendon, attached to the middle phalanx, flexes the PIP joint, while the FDP tendon is attached to the distal phalanx and flexes the DIP joint. When the PIP joint flexes, the lateral slip of the EDC tendon loosens up and allows the DIP joint to flex. The structure of the EDC tendon limits the DIP joint range of motion, which spans from full extension to a maximum permissible flexion angle, which increases for the higher PIP angle [25] as determined by the PIP–DIP interdependence relation [26]. Finally, the MCP joint is indirectly flexed under the effects of the forces transmitted by the FDP and FDS tendons that are attached further along the kinetic chain of the finger, specifically at the ventral side of the distal and middle phalanges. The torques generated by these two tendons are transmitted between the segments of the chain until they reach the MCP joint where their effect is combined. The sheaths (Figure 1), which are responsible to keep the tendons close to the phalanges, convert the linear force in the tendons to torque at the joins.

In conclusion, the extrinsic muscle–tendon units of the hand produce the gross movements of the fingers [28]. In the human hand, both the FDP and the FDS are activated simultaneously to flex the finger [29] and, consequently, the flexion of the proximal finger joints precedes that of the distal finger joints. The EDC tendon extends the three joints of the finger simultaneously [29] without hyperextending any of them. Therefore, the flexion and extension mechanism for the soft hand exoskeleton proposed in the present study replicates the structure and action of the extrinsic muscle–tendon units to flex and extend the finger mimicking the natural motion.

### 2.1. Proposed Flexion Mechanism

Many tendon-driven mechanisms have commonly utilized one tendon per function [30,31,32], however, the tendon system has also been earlier structured to provide multiple functions, which consequently resulted in a reduction of the number of actuators required to drive the system [33]. The latter concept inspired the mechanism proposed in this study to mimic both the FDS and FDP muscle–tendon units with one flexor exotendon. The mechanism, which is an updated version from a previous design [34], uses an exotendon routed around the finger to create a differential pulley mechanism, where one end of the exotendon pulls the middle phalanx while the other end pulls the distal phalanx to perform the functions of the FDS and the FDP, respectively. This differential mechanism forces the kinetic chain of the finger to follow a flexion trajectory as long as the finger is not in contact with an object. If the finger gets in contact with an object, it allows adapting the finger posture to the shape of the object.

The exotendon was implemented using Supra wires (Fladen fishing, Varberg, Sweden), with a maximum load of 40 N and a wire diameter of 0.3 mm. Guidance bead-like elements with a hole diameter of 1.5 mm and an outer diameter of 3 mm, were used to guide the exotendon along a predefined path across the finger of a soft glove made of nylon and polyester (Outdoor Research, OR^®^, size nine). To strengthen the soft glove and to reduce the stretching of the glove’s soft fabric at the pulling points, a nonstretchable fabric was sewed around each individual phalanx. The exotendon guidance beads were then fixed on the nonstretchable fabric using a thread (liner Ø0.26 mm, Fladen Fishing, Varberg, Sweden) that can tolerate forces of up to 90 N. The guidance beads were selected to be small, and with a smooth and slippery outer surface to minimize interfering with the movement of adjacent fingers.

**Figure 2:**
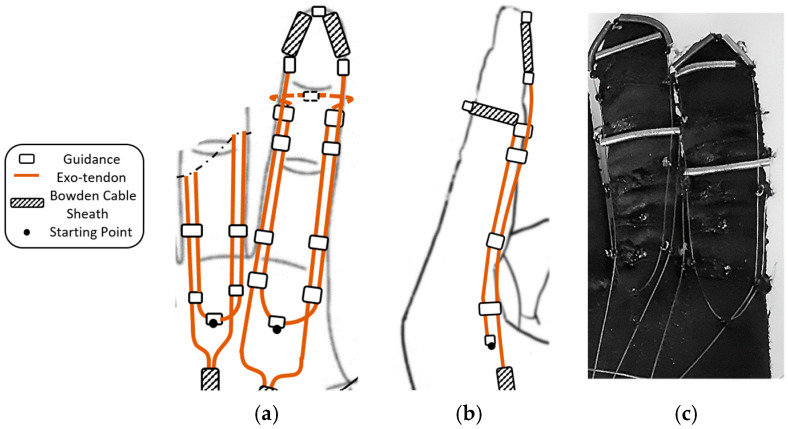
The proposed flexion mechanism, (**a**) Top view (palm side) and (**b**) Side view, including the exotendons paths, the positions of the guidance, the starting point, and the Bowden cable sheaths. (**c**) The soft glove with the mechanism implemented (palm side). Note that part of the extension mechanism is also visible.

Eight exotendon guidance beads were attached to the glove on the radial and ulnar sides of the proximal, middle, and distal phalanges, allowing the exotendon to run along the radial and ulnar sides of the finger. Two more guidance beads were located on the tip of the distal phalanx and the dorsal side of the middle phalanx (Figure 2).

The starting point of the exotendon was at the end of the first metacarpal bone before the MCP joint (Figure 2). One end of the exotendon passed through the guidance bead on the ulnar side of the finger, ascended until the middle phalanx, surrounded the distal part of the phalanx on the dorsal side, and then descended on the radial side of the finger. The other end of the exotendon passed through the guidance bead on the radial side of the finger, ascended until the distal part of the distal phalanx, and descended through the guidance bead on the ulnar side of the finger. On the distal and middle phalanges, the exotendon passed through pieces of Bowden cable sheaths to prevent the thin exotendons from being directly applied to the phalanges when pulled, which may cause discomfort. In this way, the tension force was transmitted to the Bowden cable sheaths and then to the glove fabric, which has been strengthened with a wide nonstretchable piece of fabric. 

This exotendon routing scheme allowed the mechanism to replicate the function of both FDP and FDS tendons on the finger and it was actuated by a single motor. The scheme exploited the finger to create a pulley mechanism around it. This scheme reduced the overall size of the mechanism and avoided using a pulley on the actuator side to distribute the forces between the exotendon that represented the FDP and the one that represented the FDS. 

**Figure 3:**
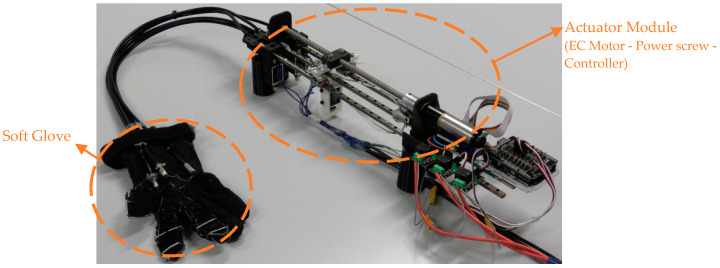
The implemented prototype. A power screw was used to transfer the power from the EC-motor to the flexion and extension mechanisms implemented on the soft glove.

The wires used as exotendons bear up to 40 N, which suffice for producing fingertip forces enough for performing activities of daily living, i.e., fingertip forces in the range of 10–15 N [13,35]. According to the relation between the tendon force and the resulting fingertip force (i.e., fingertip force = 0.35 × tendon force) [15], and taking into consideration that the proposed exotendon routing configuration pulls the finger by the two ends of the exotendon, the maximum force at the fingertip is expected to be 28 N (fingertip force = 0.35 × 2 × tendon force). Both ends of the exotendon were pulled simultaneously by the actuator, namely, an EC Maxon motor (Ø22 mm, brushless, 25 Watt) with a Planetary Gearhead (Ø22 mm, 0.5–2.0 Nm). A power screw mechanism (Ø8 mm) was coupled with the motor to convert its rotational motion to translational motion, which was used to pull the flexion mechanism (Figure 3). For finger safety, the motor was provided with a current controller to limit the maximum generated torque. This torque allowed the finger to be flexed and extended comfortably and to keep the finger at the flexed or extended position without discomfort. The maximum motor current was set at 550 mA in all tests. 

### 2.2. Proposed Extension Mechanism

The proposed extension mechanism aims at providing finger extension motion that is close to normal human motion while preventing the hyperextension of the DIP and MCP joints. The mechanism mimics the action of the extrinsic extensor muscle by using two exotendons inserted at the middle and distal phalanges that are pulled together by the same actuator. This emulated the EDC tendon, with its central and lateral slips inserted at the middle and distal phalanges, respectively, as described earlier.

**Figure 4:**
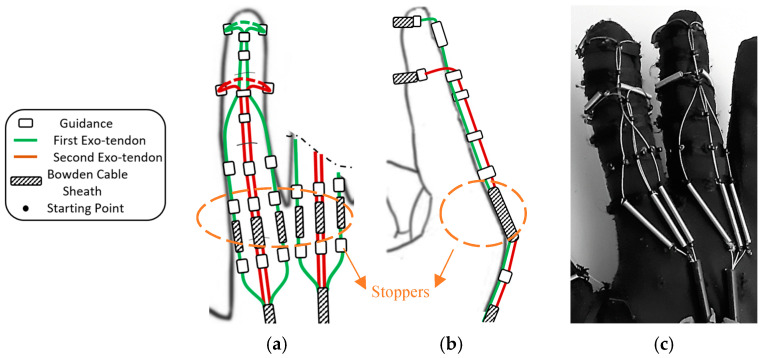
The proposed extension mechanism, (**a**) Top view (dorsal side) and (**b**) Side view, including the exotendons paths, the positions of the guidance, the starting point, the Bowden cable sheaths, and the stoppers for preventing hyperextension of the MCP joint. (**c**) The soft glove with the mechanism implemented (dorsal side).

Eight exotendon guidance beads were attached to the glove on the radial and ulnar sides of the distal, proximal, and middle phalanges, allowing the exotendons to run along the radial and ulnar sides of the finger. Six more guidance beads were located on the dorsal side of the distal, middle, and proximal phalanges (Figure 4).

Two exotendons were used to extend each finger. The first exotendon passed through a separate piece of Bowden cable on the radial and ulnar side of the MCP joint, then through the guidance bead on the radial and ulnar side of the finger, until it reached the distal aspect of the proximal phalanx (Figure 4). Then, the exotendon passed through the guidance bead on the distal aspect of the middle and distal phalanges on the dorsal side of the finger. Finally, the first exotendon was fixed on the distal phalanx by routing it around the phalanx itself using a piece of Bowden cable sheath to prevent the exotendon from being applied directly to the phalange when being pulled (Figure 4). The second exotendon passed through a separated piece of Bowden cable on the dorsal side of the MCP joint, then through the guidance bead on the dorsal side of the proximal phalanx, until reaching the distal aspect of the middle phalanx (Figure 4). Finally, the second exotendon was fixed on the middle phalanx by routing it around the phalanx using a piece of Bowden cable sheath.

The proposed scheme for the exotendons on the dorsal side of the finger (i.e., the extension side) was designed to kinematically couple the PIP and DIP joints which provided the same extension relation between the two joints every time it extended the finger. The passive joint’s stiffness did not affect the relationship. Therefore, it was expected to function in a similar manner whether the finger was not stiff or stiff (i.e., spastic finger). 

To prevent the MCP joint from hyperextending, the exotendons were routed through so-called stoppers, i.e., short Bowden cable sheaths placed on the dorsal side of the MCP joint (Figure 4a,b). The stoppers prevented the distance between the guidance beads, placed before and after the joint, to shorten further than their length. Therefore, they stopped the movement of the MCP joint at a certain angle, thereby preventing hyperextension. This was expected to function similarly whether the finger was not stiff or stiff (i.e., spastic finger).

The proposed extension mechanism was implemented using the soft glove and the actuation setup described in the previous section.

## 3. Experimental Validation of the Flexion and Extension Mechanisms

To assess the performance of the proposed mechanisms, a series of experiments were conducted with both an artificial finger and a human finger.

### 3.1. Artificial Finger

The traditional flexion and extension mechanisms, as found in the literature [17,19], insert the exotendons at the distal part of the distal phalanx, unlike the proposed mechanisms, where the exotendons are inserted at both the distal and middle phalanges. The test on an artificial finger was conducted to compare the finger kinematics while using the traditional mechanism and the proposed mechanism under the same circumstances. 

**Figure 5:**
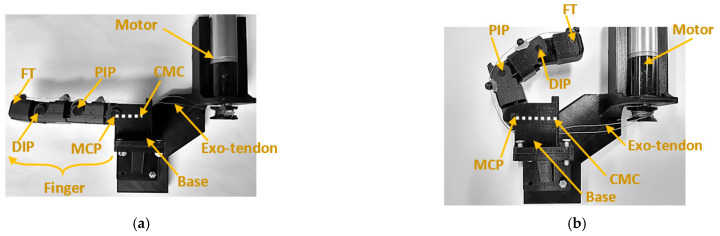
Artificial finger in its initial position for assessing (**a**) flexion and (**b**) extension. Note: the exotendons are not under tension since the artificial finger is in a relaxed state.

The artificial finger consisted of three links and a base connected to create the three joints of the finger. Torsional springs were fixed at the joints to mimic the passive joint stiffness. The reference profiles for passive joint stiffness were taken from Kamper et al. [36]. The passive joint stiffness was assumed to be zero when the finger was in the neutral position, i.e., neither flexed nor extended (DIP, PIP, and MCP angles were assumed 0.17 rad, 0.48 rad, and 0.48 rad, respectively). The stiffness increases gradually at the three joints when flexing or extending the finger from the neutral position (Table 1). The nonlinear quasi-static joint stiffness of the DIP, PIP, and MCP joints was linearized by the least square method between 0° and 90° (Table 1). The linearized stiffness values were used as the required spring stiffness constant for each joint. One set of springs was used to test the flexion mechanism (AF-F, Figure 5a, Table 1) and another for the extension (AF-E, Figure 5b, Table 1). Finally, a third set of springs (AF-ES) was implemented to mimic a spastic finger. In this case, the stiffness of the DIP, PIP, and MCP joints were 3.5, 76.9, and 54.9, respectively, which was larger than that of the AF-E. The relation between the stiffness of the three joints was therefore 3.5:76.9:54.9 (i.e., DIP:PIP:MCP), which is close to that reported for a spastic finger 0.03:0.66:0.46 [37]. 

The kinematics of the artificial finger during flexion and extension was captured using eight Qualisys Oqus 300/310 cameras and five spherical reflective markers (12.5 mm diameter) at a sampling rate of 100 Hz. The reflective markers were fixed directly over the artificial finger at the fingertip (FT), the DIP, PIP, and MCP joints, and at the base representing the carpometacarpal (CMC) joint, as shown in Figure 5a,b. The five markers were aligned when the artificial finger was fully extended (Figure 5a). The vector that represents the distal ($\bar{FT\;DIP}$), middle ($\bar{DIP\;PIP}$), and proximal ($\bar{PIP\;MCP}$) phalanges, in addition to the vector on the base ($\bar{MCP\;CMC}$) were used to measure the position of the artificial finger joints. The joint angle was measured as the angle between the vectors that represent the phalanxes just before and after the joint.

(1) Testing the flexion mechanism: The traditional and the proposed flexion mechanisms were used to flex the AF-F artificial finger, each mechanism mounted on a separate finger. The tendons were connected to a motor (Figure 5a) which, when activated, pulled on the tendons. The movement data were collected from the moment the finger started flexing from the fully extended position with the three phalanges aligned, to the maximum flexion that could be obtained by activating the mechanisms. 

(2) Testing the extension mechanism: The approach was the same as for AF-F. The movement data were collected from the moment the AF-E finger started extending from the neutral position, when DIP, PIP, and MCP joints angles were around 30°, 60°, and 60°, respectively, to the maximum extension the finger could reach using the mechanisms.

(3) Testing the extension mechanism on artificial finger simulating spasticity: The approach was the same as for AF-E, however, only the proposed mechanism was evaluated in this case. 

### 3.2. Human Finger

Due to the linearization of the passive joint stiffness and simplifying the synovial hinge joints of the human finger to pure revolution joints, the kinematics of the artificial finger might be different from that of the human finger [38]. However, these artificial fingers are only used for comparing the proposed and traditional mechanisms under the same circumstances. To evaluate the exact kinematics of the finger, actuated by the proposed mechanisms, experiments were conducted on human subjects.

Eight healthy male subjects with a hand size of nine were recruited. The glove had to exactly fit the hand to reduce the stretching of the glove’s fabric when pulling force was applied. The experiment in this section targets the assessment of the flexion/extension mechanisms implemented on the soft active glove described in Section 2, and to validate the results from the experiments with the artificial fingers. The results were compared with the voluntary motion of the middle finger of the healthy subjects. All the subjects gave written consent to participate in the experiments, which were conducted following the Declaration of Helsinki.

A custom-made, instrumented glove was implemented to measure the kinematics of the middle finger (i.e., MCP, PIP, and DIP joint angles). Three unidirectional flexible bend sensors (Spectra symbol Inc., Salt Lake City, Utah, USA), with 5.5 cm length, were attached directly to the glove, aligned with the finger, and placed on top of each joint of the finger. The angles were recorded throughout each trial, sampled at 50 Hz, and stored for later analysis.

The muscle activity from FDS and EDC was monitored during each trial to ensure that the muscles were relaxed and did not contribute actively to the finger flexion or extension. Two surface electrodes (Ambu Neuroline, Inc., Columbia, Maryland, USA), with an interelectrode distance of 2 cm, were placed along the fibers of each muscle on the anterior side of the forearm for the FDS, and the posterior side for the EDC at 2/3 of the distance from the wrist to the elbow. The reference electrode was placed at the elbow. The linear envelope of the captured EMG signals was monitored by the experimenter to ensure that it was always below the threshold (T) indicating the resting state. The threshold was defined as the mean plus three times the standard deviation of the EMG signal acquired from the respective muscle (FDS or EDC) while the subject was asked to relax the hand on the table for 10 s. 

(1) Testing the flexion mechanism: The soft active glove with the flexion mechanism implemented on it was used to flex the middle finger of the right hand. The subjects wore the instrumented glove over the soft active glove and were asked to relax the hand (monitored by observing the EMG) and place it with the ulnar aspect on the surface of a table. The middle finger was first extended using the soft glove and then the soft glove flexed the finger from this position to full flexion while the motion measurement system recorded the flexion motion. Later, the voluntary, unassisted, movement of the middle finger was measured from a fully extended position (i.e., fingers are almost straight with the palm) to a fully flexed position (i.e., hand in fist position).

(2) Testing the extension mechanism: The setup was the same as when testing the flexion. The soft glove was first activated to flex the middle finger until the end of the range of motion and then the soft glove extended the finger from this position to full extension while the motion measurement system recorded the extension motion. Later, the voluntary, unassisted, movement of the middle finger was measured from a fully flexed position to a fully extended position.

(3) Testing the adaptability of the mechanism: The aim was to assess the ability of the proposed flexion mechanism to adapt the finger posture to the shape of an object that gets in contact with the finger. The setup was the same as in the previous tests. In this experiment, the movement of the DIP joint was blocked using a finger splint to simulate a scenario, where the DIP is extended on the grasped object surface, while the PIP and MCP joints flexed to increase the area of contact between the finger and the object and to perform a firm grasp. The finger splint was fixed on the joint with its rigid brace extended along the distal and middle phalanges. After preparing the setup as described, the mechanism was activated while the motion measurement system started recording the finger flexion.

## 4. Results

### 4.1. Artificial Finger

**Figure 6:**
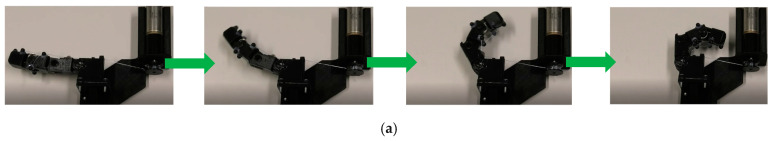

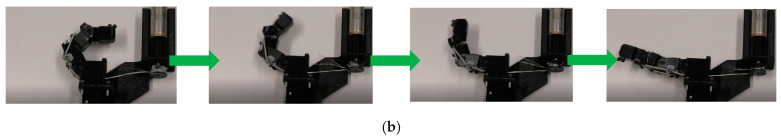
(**a**) Flexing the artificial finger (AF-F) from the extension position using the proposed flexion routing configuration. (**b**) Extending the artificial finger (AF-E) from the flexion position using the proposed extension routing configuration.

**Figure 7:**
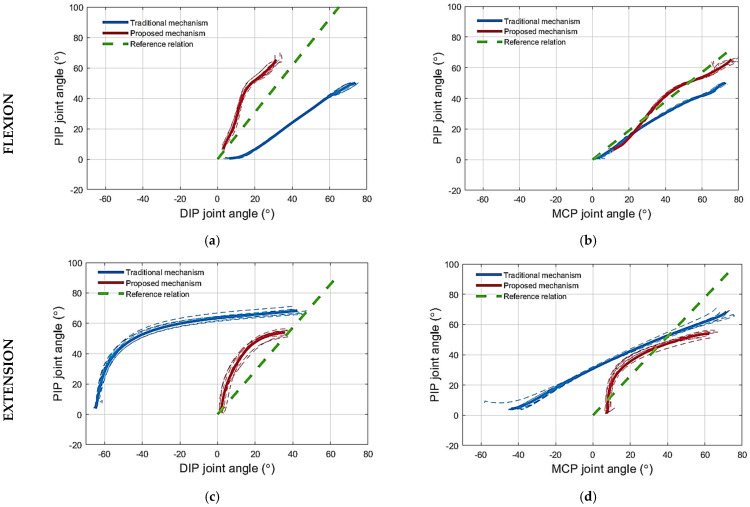
The phase plots for the finger joint angles during flexion and extension of the artificial finger using novel mechanism and traditional solution: (**a**) PIP–DIP joint angle relation during flexion; (**b**) PIP–MCP joint angle relation during flexion; (**c**) PIP–DIP joint angle relation during extension; (**d**) PIP–MCP joint angle relation during extension. Stippled lines are individual trials, and thick lines are the averages for the proposed (red) and traditional mechanism (blue). The dashed green lines are the reference inter-joint relations taken from the literature [29,39].

**Figure 8:**
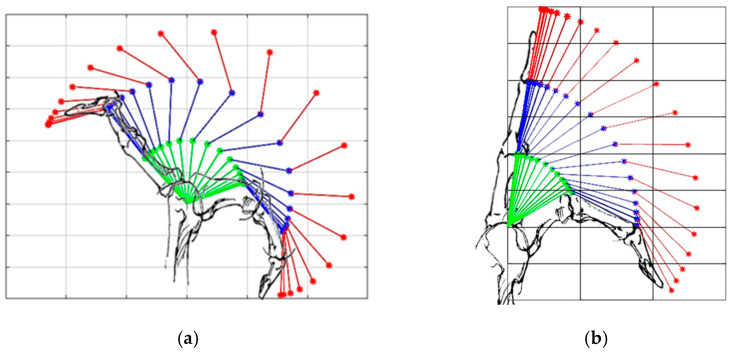
Overall finger extension (**a**) using the traditional mechanism and (**b**) using the proposed mechanism. The green line represents the proximal phalanx, the blue line represents the middle phalanx, and the red line represents the distal phalanx.

**Figure 9:**
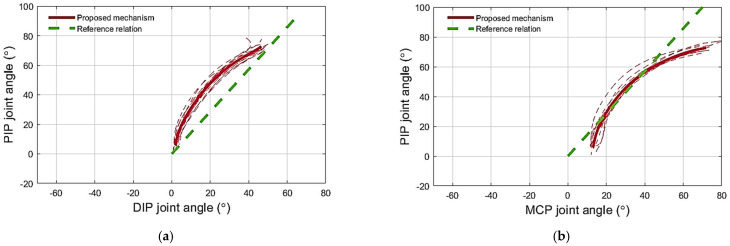
(**a**) PIP–DIP joint angle relation obtained when extending the stiff artificial finger, simulating spasticity, using the proposed extension mechanism. (**b**) PIP–MCP joint angle relation obtained when extending the stiff artificial finger, simulating spasticity, using the proposed extension mechanism. The PIP–DIP and PIP–MCP reference relations reported in the literature [29,39] for the human finger are shown as green dashed lines.

(1) Testing the flexion mechanism: The flexion of the artificial finger using the proposed routing configuration is presented in Figure 6a. During flexion, using the proposed mechanism, the DIP joint angle was consistently smaller compared to that obtained with the traditional mechanism for any PIP angle, as shown in Figure 7a. For reference, the PIP–DIP interdependence relation for the human index finger (i.e., $\theta_{DIP}=0.65*\theta_{PIP}$) [39], is also shown. Note that the proposed solution generated a profile that was much closer to the reference. Regarding the relation between the PIP and the MCP joint angle, the profile for the proposed mechanism followed closely that of the traditional mechanism, as shown in Figure 7b, until an MCP joint angle of 24°. Beyond this point, the PIP joint flexed more when using the proposed mechanism compared to the traditional mechanism, thereby approaching closer to the PIP–MCP reference relation [39,40] (i.e., $\theta_{MCP}=1.05*\theta_{PIP}$).

(2) Testing the extension mechanism: The extension of the artificial finger using the proposed routing configuration is presented in Figure 6b. When using the proposed mechanism to extend the finger, the joint angles of the artificial finger closely followed the PIP–DIP interdependence relation for the human index finger [39], without hyperextending the DIP joint, unlike the extension of the artificial finger using the traditional mechanism, as shown in Figure 7c. Regarding the relation between the PIP and the MCP joint angle, the results were similar, as the angles generated by the proposed solution followed more closely the relation modeled in the literature (i.e., $\theta_{MCP}=0.77*\theta_{PIP}$) [29], compared to the angles produced by the traditional mechanism, especially after extending the MCP joint angle below 34°, as shown in Figure 7d. The initial finger posture imposed by the proposed mechanism (i.e., the starting point of the PIP–MCP joint relation in Figure 7d) was not close to the reference finger posture (i.e., the reference PIP–MCP joint relation in Figure 7d). Therefore, during the initial phase of extension, the MCP joint extended faster than the PIP until the finger posture got close to (i.e., intersected) the reference finger extension posture. After that, the finger followed the reference relation more closely. When using the traditional mechanism, however, the generated profile deviated substantially from the reference. Finally, at the MCP angle of 7°, the finger stopped moving the MCP joint to avoid overextension. The exotendon tension force bypassed the MCP joint to the PIP joint, which continued to extend until reaching the angle of 2°. Contrary to the proposed solution, the application of the traditional mechanism led to overextension in both cases (Figure 8a,b).

(3) Testing the extension mechanism on the artificial finger simulating spasticity: The PIP–DIP joint angle relation while extending the artificial finger simulating spasticity followed closely the PIP–DIP interdependence relation for the human index finger [39], despite the joints being stiffer (Figure 9a). 

The extension mechanism prevented the DIP and MCP from hyperextending, stopping the movement at approximately 2° and 12° of flexion, respectively, as shown in Figure 9a,b.

### 4.2. Human Finger

**Figure 10:**
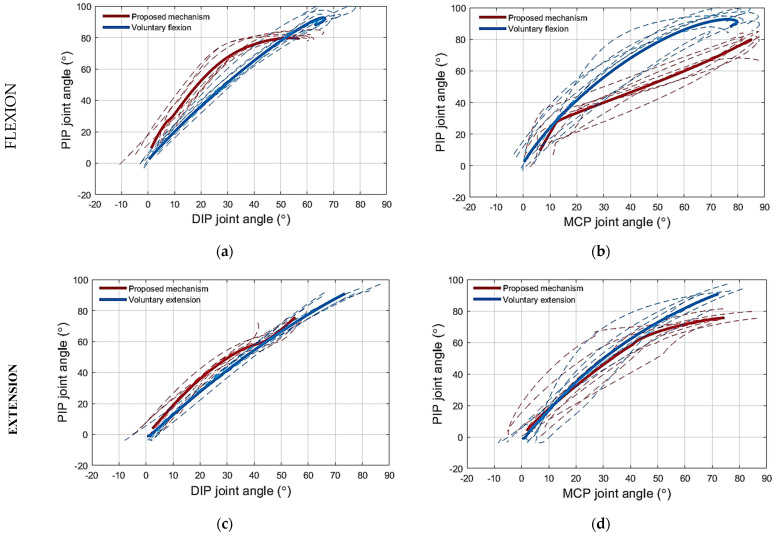
The phase plots for the finger joint angles during flexion and extension for the middle finger of healthy subjects using the novel mechanism: (**a**) PIP–DIP joint angle relation during flexion; (**b**) PIP–MCP joint angle relation during flexion; (**c**) PIP–DIP joint angle relation during extension; (**d**) PIP–MCP joint angle relation during extension. Thick lines are the averages for the proposed mechanism (red) and the voluntary motion (blue).

**Figure 11:**
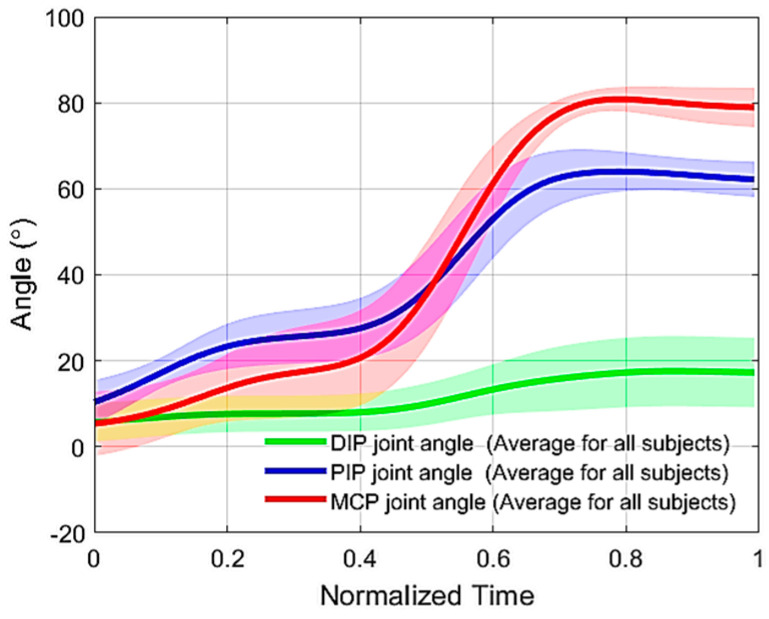
DIP, PIP, and MCP joint flexion trajectories for the middle finger of healthy subjects flexed using the proposed mechanism after blocking the DIP joint with a finger splint. The thick lines are the means, and the shaded areas represent the standard deviation.

(1) Testing the flexion mechanism: The PIP–DIP joint angle relation obtained while flexing the middle finger of the healthy subjects using the proposed flexion mechanism is shown in Figure 10a. The relation did not fully coincide with that recorded during voluntary movement (Figure 10a), however, the two profiles were close to each other. The angle of the DIP joint generated during flexion using the proposed mechanism tended to be smaller than that produced during voluntary flexion with a maximum difference of 10°. Regarding the PIP–MCP joint angle relation, the PIP joint angle recorded during voluntary movement was always larger than that obtained while flexing the finger using the proposed mechanism (Figure 10b) for any MCP angle.

(2) Testing the extension mechanism: The PIP–DIP joint angle relation when extending the middle finger of the healthy subjects with the proposed extension mechanism is shown in Figure 10c. The proposed mechanism resulted in a PIP–DIP joint angle relation similar to that recorded during voluntary movement (Figure 10c). The extension mechanism prevented the DIP and MCP joints from hyperextending. The finger followed closely the PIP–MCP joint angle relation computed from voluntary movement (Figure 10d). 

(3) Testing the adaptability of the mechanism: Blocking the DIP joint did not prevent the PIP and MCP joints from flexing (Figure 11). The latter reached 62 ± 4° and 79 ± 4° of flexion, respectively, while the DIP joint was blocked at 17 ± 7°.

## 5. Discussion

Bioinspired tendon-based underactuated mechanisms (i.e., exotendon routing configurations) for finger flexion and extension were presented in this study. Both mechanisms were inspired by the human musculoskeletal finger structure to provide close-to-normal finger motions. The mechanisms were based on the extrinsic muscle–tendon units of the hand, including the FDP and FDS muscle–tendon units for flexion and the EDC muscle–tendon unit for extension. The function of the intrinsic muscle–tendon units was partially compensated by adding stoppers to the extension mechanism at the dorsal side of the MCP joint, thereby partially emulating the function of the lumbrical muscles. The entire system employed only one actuator, reducing its overall complexity and contributing to the wearability and portability of any device implementing the proposed design, which consequently allowed the device to be used as an assistive device in daily life besides being a rehabilitation tool in the clinic. This study evaluated the impact of replicating the action of the extrinsic muscles through biomimetic routing of artificial tendons on the kinematics of the imposed finger motion during flexion and extension.

The traditional exotendon routing configuration for finger flexion, where the exotendon is inserted directly at the distal phalanx, has been used in most of the soft hand exoskeletons in the literature [14]. This configuration has been shown to result in flexion of the distal phalanx, followed by flexion of the middle and finally the proximal phalanx [14], which has been confirmed in the current study when testing the traditional flexion exotendon routing on an artificial finger. However, flexing the same artificial finger using the proposed mechanism, resulted in simultaneous flexion of the distal, middle, and proximal phalanges as it happens during natural finger flexion [14,29]. Furthermore, the DIP joint angle was always within the expected range with respect to the PIP joint angle [25,41].

The implementation of the proposed exotendon routing configuration for flexion as a pulley system allowed for the distribution of the tension force through the exo-tendon equally between the distal and the middle phalanges, thereby pulling them simultaneously. The exotendon in this configuration performed the functions of two tendons, the FDP and FDS, using one motor. 

In the human musculoskeletal system, the FDP and FDS muscle–tendon units contribute to the flexion of the PIP joint with a ratio of 1.34 to 1 [42]. The experiments conducted with the artificial finger demonstrated that flexing the finger with the proposed mechanism resulted in larger PIP joint angles than when using the traditional mechanism for the same DIP joint angles. This is because the exotendon that mimics the function of the FDS tendon is absent in the traditional mechanism. In other words, the DIP joint flexes less with the proposed mechanism compared to the traditional mechanism for the same PIP joint angles (Figure 7a). Decreasing the flexion of the DIP joint reduces the possibility of straining the DIP and PIP joint coupling mechanism [41]. These joints are coupled by the EDC tendon that spans across the two joints. The interphalangeal coupling through the EDC tendon was not simulated on the artificial finger. Therefore, the DIP joint was allowed to exceed the maximum permissible angle without any impediment. Indeed, the traditional mechanism forced the DIP joint to exceed the permissible angle of the artificial finger (Figure 7a). In the human finger, however, the excess flexion of the DIP joint will be translated into an undesired load on the EDC tendon that couples the PIP and DIP.

Regarding the relation between the PIP and MCP joints during flexion, the experiments performed on the artificial finger demonstrated that the proposed mechanism flexes the PIP joint more than the traditional mechanism for the same MCP joint angles, Figure 7b. According to the literature, such a relation holds in most of the activities of daily living [43]. Even though the PIP joint flexion angle is increased by the proposed mechanism, it is lower than the flexion angles collected during the voluntary motion (Figure 10b), for the same MCP joint angle. Nevertheless, the literature reports that the PIP–MCP joint relation is inconsistent [39] across daily life tasks due to the involvement of the intrinsic muscles, which are responsible for fine movements [28]. The PIP joint flexes more in activities that require performing, for example, a palmar grasp, especially for small and medium objects [44]. While the PIP joint angle required to perform the tripod grasp is even smaller [44]. Therefore, it is preferable that the proposed mechanism increases the PIP joint angle during flexion with respect to the MCP joint angles, however, not so much to reach the level of the PIP joint angles collected from the voluntary motion trajectories collected in this study, which present the palm grasp. 

To mimic the function of the FDP and FDS tendons, during finger flexion, using one exotendon, a pulley system was required to distribute the force equally between the two ends of the exotendon. The proposed implementation of the pulley system on the finger side of the device, Figure 2, reduced the space required if the pulley would have been implemented at the actuator side. Importantly, the proposed finger design does not change the main features of the flexion mechanism. The proposed implementation relied on routing the exotendon twice around the finger, once around the distal phalange, and then around the middle phalange using them as pulleys. The proposed solution, Figure 2, consisted thus of a compound pulley system in which the pulling force, generated by the actuator, was doubled. This feature will be assessed in future studies and it may impact the size of the actuator needed which, theoretically, could be reduced by 50% while maintaining a similar final effect on the finger.

The pulley-based differential system of the proposed flexion mechanism provides the finger with the flexibility to interact with objects in the environment. In many cases, the user grasps irregularly shaped objects, and therefore it is necessary to adapt the finger to the object surface to have a better grasp. This feature has been indeed demonstrated in the experiments by the blocking of the DIP joint during flexion to simulate the scenario of grasping objects that requires the DIP joint to be extended to increase the contact surface for a firm grasp.

Extending the finger using the traditional exotendon routing configuration was used by several soft hand exoskeletons due to its simple design [16,18]. Users might experience abnormal extension motion and joint hyperextension [19,21]. Bioinspired gloves [23,24] could provide normal finger motion, however, they required an increased design complexity. The proposed exotendon routing configuration, especially on the extension side, reduced the said complexity and kept the main function of the mechanism. It was inspired exclusively by the extrinsic muscle–tendon unit and incorporated a minor modification to compensate for the function of the lumbrical muscles, partially, i.e., a stopper was added to the exotendon routing configuration at the MCP joint to avoid the finger from being hyperextended and to pass the tension force to the PIP joint. The DIP joint did not seem to be affected by the absence of the lumbrical muscle, since the configuration provided a normal movement of the inter-phalangeal joints and prevented the DIP joint from being hyperextended.

Regarding the extension mechanism, the proposed mechanism extended the two interphalangeal joints simultaneously by coupling their motion, and thus prevented the distal phalanx from being hyperextended. The PIP–DIP joint angle traces during extension using the proposed mechanism and did not change when increasing the finger joint’s stiffness and they followed the reference finger extension profile closely (Figure 9a). This shows that, when using the mechanism, the DIP joint angle depends kinematically, but not kinetically, on the PIP joint angle. In other words, the behavior of the system did not depend on the joint stiffness and the proposed mechanism could extend both the normal and the spastic artificial finger in the same manner. The proposed mechanism also succeeded in preventing the MCP joint from being hyperextended in both the normal and spastic artificial fingers thanks to the mechanical stopper at the MCP joint (i.e., the short piece of Bowden cable mounted on top of the MCP joint). Regarding the relation between the PIP and MCP joints, the PIP–MCP joint relation produced by the proposed mechanism during extension coincides with the voluntary motion (Figure 10d). Moreover, it partially follows the reference relation modeled in the literature (Figure 7d and Figure 9b). However, the finger is not expected to follow the same path in all cases where the relationship between the PIP and the MCP joints stiffness varies significantly. The proposed mechanism provides a weak coupling between the PIP and MCP joints. The coupling depends on the stiffness of the joints, the stiff PIP joint would force the MCP joint to flex more than the PIP joint and vice versa. 

The path of the exotendons on the finger was determined by bead-like guides. These guides were selected carefully as they could have interfered with the adjacent finger and hindered its movement, which is of particular relevance when designing a multifinger system. In this study, small beads with a smooth and slippery outer surface were used. These beads-like guides showed promising results as they reduced the interference to the minimum during the experiments. However, choosing the best type of guidance requires further investigation.

To route the exotendons on the fingers, several materials and methods were reported in the literature. The HERO glove [19] used a fabric glove as a base for mounting the tendons and the guides. The Graspy glove [45] used a wide, semirigid material (e.g., VELCRO^®^) around the phalanges to strengthen a soft glove and thereby avoid stretching it when pulling with the exotendons. Liarokapis et al. [13] stitched a thin, semicircular 3D-printed structure at the phalanges with the same purpose. In the present study, to avoid applying rigid materials on the phalanges, the guides were attached to a wide nonstretchable fabric, which was stitched to the phalange of the soft glove to distribute the pulling force along the phalange, in order to obtain comfortable finger movements.

Although the proposed configuration has multiple advantages in terms of the kinematics of finger flexion and extension, its implementation has several limitations. For instance, the soft fabric of the glove was a challenge when fixing the exotendon guides and keeping them in place when applying forces. Moreover, the exotendon routing configuration for the flexion mechanism went around the finger twice to form a double pulley system, which resulted in the flexion exotendon being required to be pulled for a longer distance than required by the traditional routing configuration. This created a difference in the displacements required by the flexion exotendon to yield full finger flexion and the extension exotendon to yield full finger extension. This difference might reduce the ability to perform a smooth and instantaneous change between finger flexion and extension, causing an interruption of the finger motion, which would affect the movement frequency. Developing an actuator module to reduce this difference will be addressed in future work to enhance the frequency control which is useful for rehabilitation purposes [46]. Also, further development of the controller for the finger force may be useful for assistive applications, where it is necessary to perform grasps with proper and regulated forces [46]. In this study, the proposed flexion and extension mechanisms have been studied from a kinematics point of view. Further investigation will focus on the kinetics of the mechanism when the finger gets in contact with an object. Finally, the ability of the extension mechanism to impose the desired movement profiles independently of the joint stiffness must be tested on stroke patients.

## Figures and Tables

**Table 1:** The passive stiffness of the finger joints and its linearized stiffness for finger flexion and extension.

Joint	Finger Flexion		Finger Extension	
	Passive Joint Stiffness (Nmm/rad) ^1^	Linearized Stiffness (Nmm/rad) ^2^	Passive Joint Stiffness (Nmm/rad) ^1^	Linearized Stiffness (Nmm/rad) ^2^
$\mathrm{DIP}\;(\theta_{1}$)	$\left\{\begin{array}{cc} 0 & ,\;\theta_{1}<0.17\\ 38{\theta_{1}}^{2}-9\theta_{1}+13 & ,\;\theta_{1}\geq 0.17 \end{array}\right.$	43	$\left\{\begin{array}{cc} 38{\theta_{1}}^{2}-9\theta_{1}+13 & ,\;\theta_{1}<0.17\\ 0 & ,\;\theta_{1}\geq 0.17 \end{array}\right.$	1.1
$\mathrm{PIP}\;(\theta_{2}$)	$\left\{\begin{array}{cc} 0 & ,\;\theta_{2}<0.48\\ 106{\theta_{2}}^{2}-76\theta_{2}+40 & ,\;\theta_{2}\geq 0.48 \end{array}\right.$	56	$\left\{\begin{array}{cc} 106{\theta_{2}}^{2}-76\theta_{2}+40 & ,\;\theta_{2}<0.48\\ 0 & ,\;\theta_{2}\geq 0.48 \end{array}\right.$	5.2
$\mathrm{MCP}\;(\theta_{3}$)	$\left\{\begin{array}{cc} 0 & ,\;\theta_{3}<0.48\\ 102{\theta_{3}}^{2}-54\theta_{3}+45 & ,\;\theta_{3}\geq 0.48 \end{array}\right.$	71	$\left\{\begin{array}{cc} 102{\theta_{3}}^{2}-54\theta_{3}+45 & ,\;\theta_{3}<0.48\\ 0 & ,\;\theta_{3}\geq 0.48 \end{array}\right.$	7.4

^1^ Passive joint stiffness is the resistance to move a joint without engaging the muscles responsible for that movement. ^2^ The passive joint stiffness is linearized using least square method for simplification.

## Data Availability

Data will be made available upon request from the authors.
